# Special Issue Introduction: The GLES Open Science Challenge 2021: A Pilot Project on the Applicability of Registered Reports in Quantitative Political Science

**DOI:** 10.1007/s11615-022-00436-0

**Published:** 2022-11-21

**Authors:** Hannah Bucher, Anne-Kathrin Stroppe, Axel M. Burger, Thorsten Faas, Harald Schoen, Marc Debus, Sigrid Roßteutscher

**Affiliations:** 1 GESIS—Leibniz Institute for the Social Sciences, PO Box 12 21 55, 68072 Mannheim, Germany; 2 Freie Universität Berlin, Berlin, Germany; 3 University of Mannheim, Mannheim, Germany; 4 Goethe University Frankfurt, Frankfurt, Germany

**Keywords:** Registered reports, Open science, Quantitative political science, Electoral research, 2021 German federal election, German Longitudinal Election Study (GLES), Registered Reports, Open Science, Wahlforschung, Quantitative Politikwissenschaft, Bundestagswahl 2021, German Longitudinal Election Study (GLES)

## Abstract

The GLES Open Science Challenge 2021 was a pioneering initiative in quantitative political science. Aimed at increasing the adoption of replicable and transparent research practices, it led to this special issue. The project combined the rigor of registered reports—a new publication format in which studies are evaluated prior to data collection/access and analysis—with quantitative political science research in the context of the 2021 German federal election. This special issue, which features the registered reports that resulted from the project, shows that transparent research following open science principles benefits our discipline and substantially contributes to quantitative political science. In this introduction to the special issue, we first elaborate on why more transparent research practices are necessary to guarantee the cumulative progress of scientific knowledge. We then show how registered reports can contribute to increasing the transparency of scientific practices. Next, we discuss the application of open science practices in quantitative political science to date. And finally, we present the process and schedule of the GLES Open Science Challenge and give an overview of the contributions included in this special issue.

## Abstract (de)

Die GLES Open Science Challenge 2021 war ein Pilotprojekt in der quantitativen Politikwissenschaft, die darauf abzielte, die Akzeptanz von replizierbaren und transparenten Forschungspraktiken zu erhöhen. Dieses Sonderheft enthält die im Rahmen dieses Projektes entstandenen Artikel. Die GLES Open Science Challenge verband die strengen Regeln von Registered Reports – einem neuen Publikationsformat, bei dem Studien vor der Datenanalyse begutachtet werden – mit quantitativer Politikwissenschaft im Kontext der Bundestagswahl 2021. Die Beiträge dieses Sonderheftes zeigen, dass transparente Forschung, die den Prinzipien von Open Science folgt, unserer Disziplin zugutekommt und einen wesentlichen Beitrag zur quantitativen Politikwissenschaft leistet. In dieser Einleitung zum Sonderheft erläutern wir zunächst, warum transparentere Forschungspraktiken notwendig sind, um den kumulativen Fortschritt der wissenschaftlichen Erkenntnisse zu gewährleisten. Dann zeigen wir auf, wie Registered Reports dazu beitragen können, die Transparenz wissenschaftlicher Praktiken zu erhöhen. Anschließend diskutieren wir die bisherige Anwendung von Open-Science-Praktiken in der quantitativen Politikwissenschaft. Nachfolgend stellen wir den Prozess und den Zeitplan der GLES Open Science Challenge vor und geben abschließend einen Überblick über die in diesem Sonderheft enthaltenen Beiträge.

## Preface

The empirical social sciences are in a period of change and renewal that is characterized by efforts to improve the reliability and availability of empirical findings and—ultimately—the credibility of the theories that are based on these findings. Increasing the transparency of research practices is at the heart of many of these efforts. This involves methodological as well as cultural changes within research disciplines. Two cornerstones of these developments are preregistration and registered reports. While preregistration entails specifying and publishing hypotheses, research designs, and analysis plans prior to data collection or data access, registered reports are a publication format that takes the practice of preregistration one step further. In the registered reports model, studies are not only preregistered but also peer reviewed, revised, and accepted in principle for publication prior to data access. Hence, the editorial decision to offer in-principle acceptance of an article is based solely on the merits of the research question, research design, and analysis plan rather than on whether the empirical findings eventually turn out to be in line with predictions, ambiguous, clear, or surprising.

While the registered reports format has been increasing in popularity in various scientific disciplines in recent years, its application in quantitative political science has been tested only in a few instances. To further increase its visibility and popularity in quantitative political science, we encourage researchers and journals in our scientific discipline to consider registered reports as an appropriate publication format. To this end, we initiated the GLES Open Science Challenge 2021, a pilot project that combined the rigor of registered reports with quantitative political science in the context of the 2021 German federal election. We show that transparent research that follows open science principles is feasible and contributes to increasing the reproducibility and credibility of findings. We further demonstrate that this kind of research can make substantial contributions to our discipline.

This special issue is a compilation of the articles that resulted from the GLES Open Science Challenge 2021. All articles included in this special issue are registered reports based on German Longitudinal Election Study (GLES) data collected primarily in the weeks prior to the 2021 German federal election. The emphasis of the GLES Open Science Challenge was on the methodological innovation of facilitating the use of registered reports in quantitative political science. Given this methodological rather than thematic focus, the articles included in this special issue address a broad range of topics. Some focus on general topics recently discussed in political science, such as polarization, societal recognition, populism, and inequality, while others address topics that are directly related to the context of the 2021 German federal election, such as electoral integrity, coalition preferences, and the effects of published opinion polls.

In the following, we first illustrate the rationale for relying on registered reports to improve the quality of empirical research; we then describe the procedure of the GLES Open Science Challenge 2021; and, finally, we give an overview of the articles featured in this special issue as the outputs of this—ultimately highly successful—initiative.

## Registered Reports: A Promising Approach to Overcoming Selection Bias in Scientific Publications

Science is collaborative, cumulative, and dynamic. Theories, research methods, and paradigms are constantly being proposed, tested, and revised—and sometimes abandoned. There is a consensus that objectivity, transparency, and reproducibility are essential to science. When science follows these principles, the cumulative progress of scientific knowledge is possible. However, translating these abstract principles into concrete practices in specific scientific disciplines has been the subject of controversial debate.

One major challenge for the empirical foundation of scientific theories is the “results paradox” (Chambers and Tzavella 2022). This notion refers to the observation that academic success relies at least as much on publishing novel and statistically significant results as on applying rigorous research practices. Results-centeredness discourages researchers from submitting empirical analyses with mixed results or null findings to scientific journals and discourages journals from publishing such findings. This facilitates *publication bias*, that is, the underreporting of null results (Esarey and Wu 2016; Franco et al. 2014; Gerber and Malhotra 2008; Peplow 2014). Furthermore, it can incentivize the use of questionable research practices such as *p*-hacking, which is the post hoc adaptation of data analysis procedures in order to produce a desired result (Simmons et al. 2011), or HARKing (Banks et al. 2016; Kerr 1998), which is the formulation of hypotheses after the results are known while presenting them as if they had been generated a priori. Such research practices lead to empirical results that are not replicable by other scientists (Blaszczynski and Gainsbury 2019; Dreber and Johannesson 2019; Shrout and Rodgers 2018).

However, efforts to improve the transparency and replicability of research processes are gaining traction (Ioannidis 2005; Smith 2019). Subscribing to the open science movement, researchers are discussing how to improve the robustness of empirical research findings by increasing the transparency of research procedures. Present in many fields (Fraser et al. 2018), the movement has also reached the social sciences and, more recently, political science (Burlig 2018; Freese and Peterson 2017; Monogan 2013; Wuttke 2019).

One promising tool to overcome the results paradox and to increase the transparency of research practices is the registered reports publication format (Chambers 2013; Chambers et al. 2015, 2014; Chambers and Tzavella 2022; Nosek and Lakens 2014). In stage 1 of a two-stage publication process, authors submit a manuscript comprising an introduction, research questions and hypotheses, research design, and analysis plan, which is peer reviewed prior to data collection or access—and thus before the results are known. Stage 1 manuscripts include all parts of a typical manuscript except for the results and discussion sections. After stage 1 peer review, the manuscript is accepted in principle for publication or is rejected, or the authors are invited to revise and resubmit. As this first review round is completed before data collection or access, the analysis plan can be modified following the reviewers’ recommendations. Once in-principle acceptance has been obtained, the authors will proceed to conduct the study, adhering exactly to their peer-reviewed procedures (see Chambers 2013). After completing the paper by adding the results and discussion sections, the authors submit their final manuscript for re-review (stage 2). Subject to passing quality checks, and provided the findings are meaningfully interpreted, the manuscript is published regardless of the empirical direction, strength, or statistical significance of the results.

Registered reports have several advantages (Cook et al. 2021; Reich 2021; Reich et al. 2020; Syed and Donnellan 2020): First, hypotheses and methods must be preregistered, thereby ensuring that the decisions about data analyses are specified in stage 1 of the publication process. Divergences from preregistered procedures must be transparently documented in the resulting research. Overall, this prevents researchers from engaging in questionable research practices, such as searching for operationalizations and model specifications that support specific hypotheses or adapting hypotheses to the available data post hoc (i.e., confounding prediction and postdiction; see Nosek et al. 2018). Furthermore, it ensures that data analyses are conducted in accordance with the preregistered research plan. Second, the registered reports format entails peer review of the analysis plan before data collection or access. Peer review of an analysis plan at this early stage ensures that suggested revisions are incorporated into the data collection processes (if applicable) and the data analysis methods. Therefore, registered reports can strengthen research procedures by encouraging closer, stepwise cooperation between authors and reviewers. Third, registered reports ensure a results-blind stage 1 review and editorial decision process. As positive outcomes or significant effects cannot play a role in stage 1, registered reports help prevent publication biases (Scheel et al. 2021; Soderberg et al. 2021).

## Open Science Initiatives in Quantitative Political Science

Although registered reports improve the replicability and transparency of research practices and help avoid publication biases in scientific journals, the spread of this publication format is still relatively limited (Montoya et al. 2021). In quantitative political science, in particular, it is quite rare. However, two pilot projects aimed at improving the transparency of research practices in quantitative political science inspired the GLES Open Science Challenge 2021.

In 2015, *Comparative Political Science* (*CPS*) issued an open call for submissions of full research designs for prospective projects or already conducted projects that contained no mention of the actual results (Findley et al. 2016). Of the 19 original submissions, three were eventually granted in-principle acceptance, and, in 2016,^1^*CPS* published a special issue featuring these three articles (Bush et al. 2016; Hidalgo et al. 2016; Huff and Kruszewska 2016). Although the editors acknowledged the benefits of the results-blind review process for high-quality publications, they also observed some biases and shortcomings introduced by the publication process (Findley et al. 2016). First, submissions were biased toward quantitative research approaches (mainly survey or field experiments): “We did not receive a single qualitative submission” (Findley et al. 2016, p. 17). Second, all of the authors in the special issue were early-career scholars. In conclusion, Findley et al. (2016) noted that when implementing results-blind peer review, (1) the number of submissions with null findings may rise disproportionately because scholars might intentionally select journals that offer results-blind peer review to publish such studies; (2) circulating and discussing articles widely with colleagues is not possible, which might negatively affect the quality of submissions; and (3) scheduling reviews prior to conducting a study prolongs the research process.

The first time registered reports were applied in electoral research was in the context of the 2016 U.S. general election, when Arthur Lupia, Brendan Nyhan, and David Mellor organized the 2016 Election Research Preacceptance Competition.^2^ Scholars were invited to conduct a study on the 2016 election using American National Election Study (ANES) data and to preregister their analysis plan before the data were publicly available. Nine high-ranked political science journals took part in this initiative and agreed to review and—if deemed worthy—accept in principle registered reports submitted prior to data availability.^3^ Although more than 50 analysis plans were preregistered with a public registry, only two papers were eventually published in this competition (Enders and Scott 2019; Monogan 2020). The organizers (personal communication) suspect that the low number of publications was due to the fact that most preregistrations were submitted by early-career scholars who might have lacked experience on how to implement their ideas. In retrospect, the organizers concluded that the initiative could have benefited from early-career scholars receiving advice and mentoring from experienced researchers at the crucial stages of the process.

By now, a small number of journals in political science have adopted registered reports as a standard submission option in their journal policies. They include, for example, the *Japanese Journal of Political Science*, the *Journal of Experimental Political Science, *and* Politics and the Life Sciences.*^4^ Further, the *Journal of Politics* has announced that it will adopt registered reports as a submission format starting in January 2023.^5^ These developments and the aforementioned initiatives show that advocacy for more transparency in quantitative political science is growing. At the same time, new publication formats such as registered reports raise new questions and challenges. As mentioned above, one of the central questions is how to convince members of the scientific community across all experience levels to adopt such formats and to extend their application to a broad range of research questions and designs. Moreover, efforts are needed to familiarize researchers with the particularities of registered reports submission and review and to create opportunities for them to gain experience with the challenges, benefits, and possible limitations of this publication format. In light of these challenges, registered reports have also been referred to as a methodological innovation and part of a cultural revolution in empirical research, as researchers have to learn how to follow open science principles and apply transparent research practices (Chambers 2019).

## The GLES Open Science Challenge 2021

The GLES Open Science Challenge 2021 aimed to give quantitative political science researchers an opportunity to experience that registered reports are an appropriate and beneficial publication format for their studies. In initiating the project, we built on the experiences of the previous initiatives aimed at maximizing transparency in political science described above. By adopting the registered reports format rather than relying only on results-blind peer review, we were also able to reduce concerns raised by the editors of the *CPS* special issue (Findley et al. 2016) regarding the potential pitfalls. As both the reviewers and the authors were unaware in stage 1 of the outcomes of the analyses, intentionally submitting articles with null findings in order to get them published was not possible.^6^ On the basis of the points raised in a private communication by the organizers of the 2016 Election Research Preacceptance Competition, we consider lack of expertise in applying transparency and replicability in the research process to be one possible reason why this publication format has rarely been used so far. To reduce the hurdles for both authors and reviewers in considering registered reports as an appropriate publication format in the field of quantitative political science, we supported the authors and reviewers taking part in the GLES Open Science Challenge 2021 at every stage of the submission and review process. To this end, we implemented multiple feedback rounds throughout the process. For example, because authors had to submit an abstract prior to starting work on the registered reports (stage 0), they had already received feedback on their initial ideas and the feasibility of their analyses before embarking on stage 1. We maintained close contact with the authors and reviewers throughout the editorial process. By demonstrating that registered reports bring about substantial contributions to our discipline, we raise awareness of this publication format and contribute to ongoing efforts to make transparent research practices more popular.

### Bringing Together Registered Reports and GLES Data

All registered reports considered in the GLES Open Science Challenge 2021 rely on data from the German Longitudinal Election Study (GLES) collected in the context of the 2021 German federal election. One particularity of this project was that the organizers, who are also the editors of this special issue, were at the same time part of the GLES team responsible for the conceptualization of the study and for administering the collection, documentation, and publication of GLES data. The overlap of simultaneously being editors and data providers allowed us to coordinate the processes of data collection and data publication with the different stages of the GLES Open Science Challenge. This in turn enabled a fast-track publication process, with only approximately 12 months between abstract submission and publication of the final manuscripts. The fact that the organizers were very familiar with the data sets used by the authors to conduct their studies also allowed for providing in-depth support in setting up the registered reports. The decision to use GLES data for a preregistration challenge stemmed from a desire to show that this publication format can also be applied to secondary data. Although registered reports are most often applied in experimental contexts or to data collected for one specific study (van der Akker et al. 2019), our project shows that large surveys are also suitable for the application of transparent research practices. Given that the pre-election data are freely available to the scientific community and are released shortly after election day, and that the surveys capture a wide range of concepts and survey instruments to study political attitudes and behavior, the GLES is very appropriate for conducting an open science challenge. As data providers, the organizing team was, of course, also interested in incentivizing the use of GLES data by the scientific community. This challenge helps GLES gain visibility in quantitative political science, shows that the GLES data are suitable for different research purposes, and encourages researchers to use these data for their research. For the GLES Open Science Challenge 2021, researchers could choose between two different data sets, the GLES Pre-Election Cross-Section or the GLES Rolling Cross-Section.

#### The GLES Pre-Election Cross-Section

The GLES Pre-Election Cross-Section (GLES 2022a) is a data source that is very well suited to analyzing the voting behavior of the German electorate. The survey includes a wide range of questions on the political attitudes and behavior of the respondents, as well as on their sociodemographic characteristics. Striving for a temporally and internationally comparative perspective, many questions included in this survey are based on established concepts used in electoral research. However, the GLES also adopts innovative and current perspectives of international election and attitudinal research by including new instruments. For example, the 2021 Pre-Election Cross-Section included questions about electoral-integrity beliefs and perceived societal recognition.

The GLES Pre-Election Cross-Section (GLES 2022a) is a probabilistic survey fielded in the four weeks prior to election day. Because of the COVID-19 pandemic, the 2021 survey was conducted in a self-administered mixed-mode design consisting of computer-assisted web interviews and paper-and-pencil interviews. Ultimately, 5116 at least partial interviews were conducted between August 26 and September 25, 2021. Of those, 3716 (72.63%) respondents (aged 16 years and older) participated via web, while 1400 (27.37%) respondents completed the paper-and-pencil questionnaire.

#### The GLES Rolling Cross-Section

The GLES Rolling Cross-Section (GLES 2022b) is designed to record cross-sectional developments in political attitudes and behavior during the electoral campaign period. It is a probabilistic population survey based on computer-assisted telephone interviews that is divided into structurally equivalent daily samples according to a rolling cross-section (RCS) design (Johnston and Brady 2002). The design enables campaign-induced changes in public opinion to be captured. Voter reactions to events during the campaign can be registered, and the decay or stability of these effects can be revealed. Because the RCS study includes a post-election panel wave of the survey, it is also possible to investigate intraindividual changes between the two waves.

The pre-election data collection took place during the final 55 days of the run-up to election day and was complemented by a post-election panel wave in which available respondents from the pre-election interviews were re-interviewed. The gross sample of telephone numbers comprised 60% landline numbers and 40% mobile numbers drawn from sampling frames of the Arbeitsgemeinschaft Deutscher Markt- und Sozialforschungsinstitute. The exact number of telephone numbers to be entered into the system was determined daily, considering the current dynamics of the field period. In total, 7068 complete interviews were conducted in the pre-election period (a daily average of 128.51 interviews); 4446 (62.9%) of the pre-election respondents also completed the post-election survey.^7^

In addition to questions on key aspects of electoral research, special features of the 2021 GLES Rolling Cross-Section survey were questions with a focus on perceptions and assessments of the candidates for the chancellorship; questions to capture media use, especially reception of the TV debates; and questions related to the use of absentee voting.

### Procedure

**Fig. 1:**
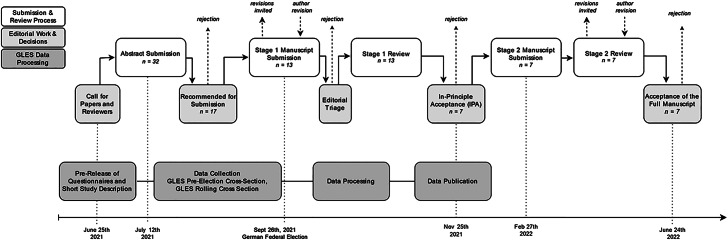
Stages of the GLES Open Science Challenge 2021

As outlined above, the motivation behind this special issue was to encourage researchers to consider registered reports as an appropriate publication format for their studies. Further, all registered reports featured in this special issue rely on survey data provided by GLES. To combine the rigor of registered reports with GLES data, we had to align data collection and preparation schedules with the submission and review processes of the GLES Open Science Challenge. Specifically, first, we published questionnaires and study documentation of the GLES surveys before the data were collected in order to give participating researchers enough time to set up their studies and become familiar with the data sets. Second, we aligned the submission and review processes with the data collection and publication processes to allow for a results-blind preparation and review of stage 1 registered reports. Third, we scheduled submission and review processes around the 2021 German federal election. Fig. 1 gives an overview of the coordination of the scheduling of the data collection and provision steps with the GLES Open Science Challenge submission and review processes.

#### Publication of Questionnaires and Study Documentation

To facilitate the preparation of registered reports, the GLES team published the questionnaires and study documentation of the GLES Pre-Election Cross-Section and the GLES Rolling Cross-Section surveys more than a month before the start of data collection. The published information included questionnaires, codebooks, survey design information, and the targeted sample size. In anticipation of unforeseeable developments during the campaign, and pending the results of pretests, the complete set of questions was not provided. Instead, the questionnaires included several placeholders. All information was published in English and was freely accessible on the GLES homepage. In addition, a fixed date for the data release was specified. This was necessary for scheduling the first round of reviews and implementing any revisions resulting from the reviews before the data were published.

#### Submission of Abstracts

In stage 0 of the GLES Open Science Challenge, we called for submissions of open topic abstracts providing a general overview of the planned study. Authors who submitted an abstract received editorial feedback on the feasibility and suitability of their contribution for the special issue. Because of coordination of the scheduling of the submission and review process with data collection and publication, abstracts had to be submitted by July 12, 2021, and thus over two months before the 2021 German federal election on September 26. As the questionnaires were published in late June, the authors had only three weeks to familiarize themselves with the questions, to come up with a research question related to the questionnaires, and to prepare and submit an abstract. We received 32 abstracts from 58 authors, which can be seen as a sign of considerable interest in the GLES Open Science Challenge.

As the abstracts had to be submitted at a time when the election campaign had not reached its political climax, the submissions covered a wide range of research questions related to elections, political attitudes, and behavior rather than focusing on the particularities of the 2021 election. Of the 32 study proposals submitted, 17 received positive feedback, and the authors were encouraged to submit a stage 1 manuscript.

#### Stage 1 Manuscript Submission and Review

Following the initial feedback on their planned studies, researchers had two months to prepare their stage 1 manuscripts. Besides standard criteria of article evaluation, the subsequent evaluation of the stage 1 manuscript submissions focused on whether the proposed analyses offered an adequate and appropriate test of the preregistered hypotheses.^8^ To enable us to align the submission of stage 1 manuscripts with the collection of GLES survey data, authors had to submit their stage 1 manuscript by September 25, 2021—the day before election day, which was also the last day of data collection for the pre-election surveys. The period between the last day of data collection for the pre-election surveys and the publication of datasets was two months. Thus, the review process of stage 1 manuscripts was scheduled in such a way that it allowed authors to incorporate reviewers’ feedback into their preregistration before accessing the data in November/December 2021. We received 13 stage 1 manuscripts from 23 authors. Seven manuscripts were accepted in principle for publication, and the authors were invited to resubmit their articles as stage 2 manuscripts after data publication and completion of the preregistered analyses.

#### Reviewer Recruitment and Provision of Information

The strict timeline of the GESIS Open Science Challenge resulted in tightly scheduled review rounds. On average, the reviewers had only two weeks to review the stage 1 manuscripts. To ensure that the review process would work under these constraints, a call for reviewers was published well in advance of the review process. The recruitment of the reviewers in that way was highly successful. By the time the stage 1 manuscripts were submitted, we had already recruited a large pool of reviewers with expertise in different subfields of electoral research, with different degrees of experience in the academic system, and from many countries. We were overwhelmed by the breadth of the response and the great willingness of scholars to participate in the GLES Open Science Challenge as reviewers. As we assumed that neither the authors nor the reviewers would have much experience with registered reports, we provided guidelines on our homepage^9^ that addressed the particularities of this article format from an author’s and a reviewer’s perspective.

#### Stage 2 Manuscript Submission and Review

Once the data were published, authors were invited to submit their stage 2 manuscripts, including the data analysis, reporting of results, and discussion. Additionally, authors were required to submit detailed analysis scripts to make each step of data cleaning, preparation, and analysis replicable. In stage 2, submissions were reviewed with regard to the implementation of the preregistered analysis plan, the interpretation of the results, and the discussion. All seven stage 1 manuscripts that obtained in-principle acceptance were resubmitted as stage 2 manuscripts and are now part of this special issue.

## The GLES Open Science Challenge: Participating Authors

Restricting submissions to our special issue to research that used GLES data may have limited the diversity of participating scholars. Because researchers already familiar with GLES data had to invest less effort in familiarizing themselves with the data, they may have been more likely to submit their ideas.^10^ We tried to overcome this risk of possible in-group bias by circulating the call for papers for the GLES Open Science Challenge through various channels and by encouraging researchers from different subdisciplines of political science and across different countries to participate. Nonetheless, it is important to take a look at some background information on the participating authors.

Table 1 provides key characteristics of participating authors at the different stages of the challenge—namely, their gender, academic status, (former) membership on the GLES team, and (former) affiliation with the University of Mannheim.^11^ Although we aimed to encourage the participation of a broad and diverse range of researchers, we have to acknowledge that many of the participating authors had some proximity to the GLES through their current or former affiliations. Further, we observe a low (and over submission stages decreasing) share of professors and female researchers. While it is difficult to say whether and to what extent this observation indicates a problem, similar future projects should invest efforts in attracting researchers without prior links to the institution running the project, researchers of different career levels, and researchers from a broad diversity of backgrounds.

**Table 1:** Key characteristics of the participating authors in the GLES Open Science Challenge 2021

		Stage 0: abstract submission	Stage 1 manuscript submission	Stage 2 manuscript submission
*Gender*	Female	31%	17%	14%
	Male	69%	83%	86%
*Academic status*	Doctoral researcher	40%	39%	43%
	Postdoctoral researcher	43%	47%	50%
	Professor	17%	13%	7%
*German affiliation*	Yes	78%	70%	79%
*(Former) member of GLES team*	Yes	16%	9%	7%
*(Formerly) Affiliated with the University of Mannheim*	Yes	52%	43%	64%
*Total*	*N*	58	23	14

## The GLES Open Science Challenge 2021: Contributions

With the empirical analyses based on the two GLES data sets described in the previous sections, this special issue brings together a diverse set of studies on relevant questions during political change and crises in the context of the 2021 German federal election. Election years not only allow for analyzing electoral behavior, short-term developments, and campaign dynamics, but they also present an opportunity to monitor (the polarization of) opinions across issues, populist sentiments, and behavioral implications of attitudes and beliefs. This diversity of analytical approaches is also reflected in the contributions in this special issue, which address various aspects of political attitudes and behavior in the 2021 German federal election.

The first three articles address topics related to electoral behavior in the context of the 2021 German federal election. Robert Welz investigated the impact of issue distance, intracoalition heterogeneity, and salience on voters’ coalition preferences (Welz 2022). Fabienne Unkelbach, Melvin John, and Vera Vogel focused on the role of voters’ social class in poll effects in the 2021 German federal election (Unkelbach et al. 2022). And Christian Schnaudt assessed the behavioral implications of electoral-integrity beliefs in Germany (Schnaudt 2022).

The remaining four articles deal with political attitudes more broadly. The first two studies are aimed at describing and analyzing populist attitudes. While Nils Steiner, Christian Schimpf, and Alexander Wuttke assessed the multiple roots of populism in feelings of lacking societal recognition (Steiner et al. 2022), Robert Huber, Michael Jankowski, and Carsten Wegscheider investigated the impact of policy discontent and representation on populist attitudes (Huber et al. 2022). The final two studies explain attitudes toward two different issues in depth: Jan Menzner and Richard Traunmüller assessed why citizens think they cannot speak freely (Menzner and Traunmüller 2022), and Denis Cohen investigated the context effects of rent control preferences (Cohen 2022).

The GLES Open Science Challenge 2021 represents the first collection of registered reports published in the *German Political Science Quarterly* (*PVS*). With this special issue, *PVS* joins the growing number of scientific journals across different disciplines that publish articles in the registered report format, which reached 278 in June 2021 (Montoya et al. 2021). The articles in this special issue are testament to the fact that the application of registered reports in quantitative political science is feasible and desirable and that it results in relevant contributions to our discipline.

## Footnotes

1 For detailed information on this initiative, see http://www.ipdutexas.org/cps-transparency-special-issue.html.

2 For detailed information on this initiative, see https://cps.isr.umich.edu/project/the-2016-election-research-preacceptance-competition/.

3 For a list, see osf.io/rr.

4 For a list, see osf.io/rr.

5 https://twitter.com/The_JOP/status/1544712044594724864.

6 One criticism often raised against registered reports is that this publication format might encourage authors to test particularly risky or novel hypotheses that are highly unlikely to be confirmed in empirical analyses. However, previous literature does not indicate that this is current practice (Soderberg et al. 2021; Scheel et al. 2021).

7 For detailed information on the study design, see the study description (GLES 2022b).

8 For a detailed description of the requirements for submitting stage 1 manuscripts, see our guidelines for authors: https://osf.io/uvnp7/.

9 https://gles.eu/gles-open-science-challenge-2021/downloads/. All materials are also available on OSF: https://osf.io/uvnp7/

10 We guarantee that none of the authors had access to unpublished data from pretests or other preliminary data sets before the actual data collection, even if they are affiliated or were formerly affiliated with the GLES team.

11 Like a number of other German institutions, the University of Mannheim has had strong ties with GLES for many years. However, affiliation with the University of Mannheim does not necessarily imply experience with GLES data or a connection to the data collection processes.

## References

[CR1] Banks GC, Rogelberg SG, Woznyj HM, Landis RS, Rupp DE (2016). Editorial: Evidence on questionable research practices: the good, the bad, and the ugly. Journal of Business and Psychology.

[CR2] Blaszczynski A, Gainsbury SM (2019). Editor’s note: replication crisis in the social sciences. International Gambling Studies.

[CR3] Burlig F (2018). Improving transparency in observational social science research: A pre-analysis plan approach. Economics Letters.

[CR4] Bush SS, Erlich A, Prather L, Zeira Y (2016). The effects of authoritarian iconography. Comparative Political Studies.

[CR5] Chambers CD (2013). Registered Reports: A new publishing initiative at Cortex. Cortex.

[CR6] Chambers CD (2019). The registered reports revolution: lessons in cultural reform. Significance.

[CR9] Chambers CD, Tzavella L (2022). The past, present, and future of Registered Reports. Nature Human Behaviour.

[CR7] Chambers CD, Feredoes E, Muthukumaraswamy SD, Etchells P (2014). Instead of “playing the game” it is time to change the rules: Registered Reports at AIMS Neuroscience and beyond. AIMS Neuroscience.

[CR8] Chambers CD, Dienes Z, McIntosh RD, Rotshtein P, Willmes K (2015). Registered Reports: realigning incentives in scientific publishing. Cortex.

[CR48] Cohen, Denis. 2022. Preferences for Rent Control: Between Political Geography and Political Economy. *Politische Vierteljahresschrift*. 10.1007/s11615-022-00404-8.

[CR10] Cook BG, Maggin DM, Robertson RE (2021). Registered reports in special education: introduction to the special series. Remedial and Special Education.

[CR11] Dreber A, Johannesson M, Dreber A, Johannesson M (2019). Statistical significance and the replication crisis in the social sciences. Oxford research encyclopedia of economics and finance.

[CR12] Enders AM, Scott JS (2019). The increasing racialization of American electoral politics, 1988–2016. American Politics Research.

[CR13] Esarey J, Wu A (2016). Measuring the effects of publication bias in political science. Research & Politics.

[CR14] Findley MG, Jensen NM, Malesky EJ, Pepinsky TB (2016). Can results-free review reduce publication bias? The results and implications of a pilot study. Comparative Political Studies.

[CR15] Franco A, Malhotra N, Simonovits G (2014). Publication bias in the social sciences: unlocking the file drawer. Science.

[CR16] Fraser H, Parker T, Nakagawa S, Barnett A, Fidler F (2018). Questionable research practices in ecology and evolution. PloS ONE.

[CR17] Freese J, Peterson D (2017). Replication in social science. Annual Review of Sociology.

[CR18] Gerber A, Malhotra N (2008). Do statistical reporting standards affect what is published? Publication bias in two leading political science journals. Quarterly Journal of Political Science.

[CR19] German Longitudinal Election Study (GLES) (2022). GLES Querschnitt 2021, Vorwahl (ZA7700 Datenfile Version 2.0.0).

[CR20] German Longitudinal Election Study (GLES) (2022). GLES Rolling Cross-Section 2021 (ZA7703 Data File Version 2.0.0).

[CR21] Hidalgo FD, Canello J, Lima-de-Oliveira R (2016). Can politicians police themselves? Natural experimental evidence from Brazil’s Audit Courts. Comparative Political Studies.

[CR46] Huber, Robert A., Michael Jankowski, and Carsten Wegscheider. 2022. Explaining Populist Attitudes: The Impact of Policy Discontent and Representation.* Politische Vierteljahresschrift.*10.1007/s11615-022-00422-6.

[CR22] Huff C, Kruszewska D (2016). Banners, barricades, and bombs. Comparative Political Studies.

[CR23] Ioannidis JPA (2005). Why most published research findings are false. PLoS Medicine.

[CR24] Johnston R, Brady HE (2002). The rolling cross-section design. Electoral Studies.

[CR25] Kerr NL (1998). HARKing: hypothesizing after the results are known. Personality and Social Psychology Review.

[CR47] Menzner, Jan, and Richard Traunmüller. 2022. Subjective Freedom of Speech: Why Do Citizens Think They Cannot Speak Freely? *Politische Vierteljahresschrift*. 10.1007/s11615-022-00414-6. 10.1007/s11615-022-00414-6 PMC9368691 35971507

[CR26] Monogan JE (2013). A case for registering studies of political outcomes: an application in the 2010 House elections. Political Analysis.

[CR27] Monogan JE (2020). Anxious voters in the 2016 U.S. election: an analysis of how they decided from the ERPC2016. Political Behavior.

[CR28] Montoya AK, Krenzer WLD, Fossum JL (2021). Opening the door to registered reports: census of journals publishing registered reports (2013–2020). Collabra: Psychology.

[CR29] Nosek BA, Lakens D (2014). Registered reports. Social Psychology.

[CR30] Nosek BA, Ebersole CR, DeHaven AC, Mellor DT (2018). The preregistration revolution. Proceedings of the National Academy of Sciences of the United States of America.

[CR31] Peplow M (2014). Social sciences suffer from severe publication bias. Nature.

[CR32] Reich J (2021). Preregistration and registered reports. Educational Psychologist.

[CR33] Reich J, Gehlbach H, Albers CJ (2020). “Like upgrading from a typewriter to a computer”: registered reports in education research. AERA Open.

[CR34] Scheel AM, Schijen MRMJ, Lakens D (2021). An excess of positive results: comparing the standard psychology literature with registered reports. Advances in Methods and Practices in Psychological Science.

[CR44] Schnaudt, Christian. 2022. Exit or Voice? Behavioral Implications of Electoral-Integrity Beliefs in Germany. *Politische Vierteljahresschrift*. 10.1007/s11615-022-00403-9.

[CR35] Shrout PE, Rodgers JL (2018). Psychology, science, and knowledge construction: broadening perspectives from the replication crisis. Annual Review of Psychology.

[CR36] Simmons JP, Nelson LD, Simonsohn U (2011). False-positive psychology: undisclosed flexibility in data collection and analysis allows presenting anything as significant. Psychological Science.

[CR37] Smith, Noah. 2019. Why economics is having a replication crisis: Recreating research by gathering data from the real world and analyzing it statistically often fails to produce the same result. *Bloomberg Opinion*. https://www.bloomberg.com/view/articles/2018-09-17/economics-gets-it-wrong-because-research-is-hard-to-replicate. Accessed: September 19, 2019.

[CR38] Soderberg CK, Errington TM, Schiavone SR, Bottesini J, Singleton Thorn F, Vazire S, Esterling KM, Nosek BA (2021). Initial evidence of research quality of registered reports compared with the standard publishing model. Nature Human Behaviour.

[CR39] Syed M, Donnellan MB (2020). Registered reports with developmental and secondary data: some brief observations and introduction to the special issue. Emerging Adulthood.

[CR43] Unkelbach, Fabienne, Melvin John, and Vera Vogel. 2022. Jumping on the Bandwagon: The Role of Voters’ Social Class in Poll Effects in the Context of the 2021 German Federal Election. *Politische Vierteljahresschrift.*10.1007/s11615-022-00417-3. 10.1007/s11615-022-00417-3 PMC9364306 35967251

[CR40] van den Akker, Olmo, Sara J. Weston, Lorne Campbell, William J. Chopik, Rodica I. Damian, Pamela Davis-Kean, Andrew Nolan Hall, Jessica Elizabeth Kosie, Elliott Tyler Kruse, Jerome Olsen, Stuart James Ritchie, Kathrene D. Valentine, Anna Elisabeth van ’t Veer, and Marjan Bakker. 2019. *Preregistration of secondary data analysis: a template and tutorial.* PsyArXiv. https://psyarxiv.com/hvfmr/.

[CR42] Welz, Robert. 2022. At Least Agree on the Important Things: The Impact of Issue Distance, Intracoalition Heterogeneity, and Salience on Voters’ Coalition Preferences. *Politische Vierteljahresschrift*. 10.1007/s11615-022-00415-5.

[CR41] Wuttke A (2019). Why too many political science findings cannot be trusted and what we can do about it: a review of meta-scientific research and a call for academic reform. Politische Vierteljahresschrift.

[CR45] Steiner, Nils. D., Christian. H. Schimpf, and Alexander Wuttke. 2022. Left Behind and United by Populism? Populism’s Multiple Roots in Feelings of Lacking Societal Recognition. *Politische Vierteljahresschrift.*10.1007/s11615-022-00416-4.

